# The impact of post thaw embryo culture (extended culture) on frozen embryo transfer outcomes: a systematic review and meta-analysis

**DOI:** 10.1007/s10815-026-03919-w

**Published:** 2026-06-12

**Authors:** Adva Cahen-Peretz, Lilah Tsaitlin-Mor, Tomer Ben-Shushan, Hagai Levine, Anat Hershko-Klement, Yaakov Bentov

**Affiliations:** 1 Obstetrics and Gynecology department, Hadassah Mount Scopus medical center and Faculty of Medicine, Hebrew University of Jerusalem, Jerusalem, Israel; 2 Faculty of Medicine, Hebrew University of Jerusalem, Jerusalem, Israel; 3 Braun School of Public Health and Community Medicine, Hebrew University of Jerusalem, Jerusalem, Israel; 4 Faculty of Medicine, Hebrew University Medical Center, Jerusalem, Israel

**Keywords:** Frozen embryo transfer, Post-thaw culture, Vitrification, Embryo development, Blastocyst culture

## Abstract

**Purpose:**

To systematically evaluate the impact of post-thaw culture duration on frozen embryo transfer (FET) outcomes across different embryonic developmental stages.

**Methods:**

We conducted a systematic review and meta-analysis following PRISMA. A comprehensive search of Medline, Embase, Web of Science, and Cochrane identified 14 eligible studies (*N* = 17,415) utilizing vitrification. The analysis investigated four post-thaw protocols (extended culture 18–48 h), stratified by embryonic developmental stage: (A) D2ext: day 2 extended vs. day 2 (3 studies, *N* = 879), (B) D3ext: day 3 extended vs. day 3 (3 studies, *N* = 11,204), (C) D3-to-D5/6: day 3 extended to day 5/6 vs. day 5/6 (4 studies, *N* = 1449), and (D) D5/6ext: day 5/6 extended to day 5/6 (4 studies, *N* = 3883). Pooled relative risks (RRs) with 95% confidence intervals (CIs) and heterogeneity were calculated using random-effects models.

**Results:**

Extended culture significantly improved outcomes for day-3 embryos. D3ext increased live birth rates (RR 1.076, 95% CI 1.034–1.119), while D3-to-D5/6 showed the most pronounced benefit (RR 1.229, 95% CI 1.008–1.499) and reduced biochemical pregnancy loss. Conversely, D2ext showed significantly lower clinical pregnancy rates than immediate transfer (RR 0.689, 95% CI 0.569–0.835). For established blastocysts (D5/6ext), extended culture provided no significant improvement in live birth rates.

**Conclusion:**

Post-thaw culture strategies should be tailored to embryonic developmental stage. Our most notable finding indicates that day-2 embryos achieve significantly better outcomes with immediate transfer (2–6 h) rather than prolonged in vitro culture. Conversely, day-3 embryos benefit significantly from extended culture (18–48 h), particularly when cultured to the blastocyst stage. Extended culture offers no clinical advantage for embryos already at the blastocyst stage. Post-thaw culture strategies should therefore be strictly tailored to the specific embryonic developmental stage at the time of cryopreservation.

**Supplementary Information:**

The online version contains supplementary material available at 10.1007/s10815-026-03919-w.

## Introduction

Frozen embryo transfer (FET) has emerged as an essential element of modern assisted reproductive technology, with FET cycles comprising an increasing proportion of all embryo transfers globally [1]. This shift reflects numerous clinical advantages, including reduced ovarian hyperstimulation syndrome risk, improved embryo-endometrial synchronization, and elimination of supraphysiological hormonal environments that may compromise implantation [2, 3]. The widespread adoption of vitrification, which demonstrates significantly higher post-thaw survival rates compared to slow freezing methods, has further accelerated this transition [4–7].

While contemporary practice increasingly favors blastocyst-stage cryopreservation [8–10], substantial inventories of cleavage-stage embryos frozen at day 2–3 remain in fertility centers worldwide. These embryos present a critical clinical dilemma: should they undergo immediate transfer after thawing, or be subjected to extended post-thaw culture to more advanced developmental stages? The biological response following thawing varies significantly by developmental stage. Blastocysts typically re-expand within 1–3 h and demonstrate established developmental competence, while cleavage-stage embryos require longer observation periods to assess developmental progression and recovery from cryopreservation-induced cellular stress [11, 12]. Furthermore, while extended culture to the blastocyst stage can increase cumulative live birth rates in good prognosis patients, it is also associated with potential drawbacks, such as a higher risk of cycle cancellation and an increased incidence of premature birth [13]. This has led to considerable practice variation, with post-thaw protocols ranging from brief assessment (2–6 h) to extended culture (18–48 h) [14].

Existing research yields conflicting results regarding optimal post-thaw culture duration. Some studies report significant benefits from extended culture for certain embryonic stages, demonstrating improved implantation and pregnancy rates through enhanced embryo selection [15–20]. Conversely, other investigations show no improvement or potentially detrimental effects [21–23], suggesting that prolonged in vitro culture may compromise embryo viability.

The clinical significance extends beyond immediate pregnancy outcomes. Suboptimal protocols could unnecessarily prolong treatment cycles and increase healthcare costs, while optimized stage-specific strategies could maximize utilization of existing embryo inventories and improve cumulative pregnancy rates. Mathematical modeling approaches have been proposed to balance quantity versus quality trade-offs [24, 25] but require comprehensive evidence regarding culture duration impacts across developmental stages.

Given the substantial number of cleavage-stage embryos currently preserved worldwide, this systematic review and meta-analysis systematically evaluates the impact of extended versus immediate post-thaw culture on FET outcomes, stratified by embryonic developmental stage. Our primary objective is to provide a comparison of short versus extended culture on FET outcomes for embryos at different developmental stages (day 2, day 3, and blastocyst stage). Through this stage-specific approach, we aim to contribute evidence-based insights that may inform clinical decision-making and protocol optimization in contemporary FET practice.

## Methods

### Information sources and search strategy

A systematic review and meta-analysis investigating the influence of post-thaw embryo culture on frozen embryo transfer (FET) outcomes was conducted using an a priori protocol (registered on PROSPERO CRD42025635380) following the PRISMA guidelines for reporting systematic reviews and meta-analyses. The following deviations from the registered protocol were made: (1) Effect measures were reported as relative risks rather than odds ratios for implantation, clinical pregnancy, and live birth outcomes, as relative risks are more readily interpretable for common outcomes, while odds ratios were retained for rarer outcomes (miscarriage, embryo loss); (2) the database search encompassed 1983–2024 rather than starting from 2012 as originally specified, though in practice only studies using vitrification (predominantly from 2010 onwards) were included, consistent with our eligibility criteria; (3) Comprehensive Meta-Analysis version 4.0 was used instead of version 3.0. We conducted a comprehensive literature search using PubMed/Medline, Embase, Web of Science, and Cochrane databases from January 1, 1983, through October 28, 2024. Our research team included two REIs (reproductive endocrinologists and infertility specialists) both with knowledge in statistics, two additional gynecologists, a qualified medical librarian specializing in systematic reviews, and an epidemiologist with expertise in meta-analyses.

For the search strategy, we used combinations of keywords and controlled vocabulary related to post-thaw culture, embryo culture, frozen embryo transfer, and related outcomes. The search algorithm followed the structure: (Cryopreservation OR Freezing OR Vitrification) AND (Embryo culture technique OR Embryo Development) AND (Post thaw embryo culture) AND (Implantation OR Pregnancy rate OR Live birth rate). Specific search terms included variations of “cryopreservation,” “vitrification,” “embryo culture,” “post-thaw,” “embryo implantation,” “pregnancy rate,” and “live birth rate,” along with appropriate MeSH and EMTREE terms. Reference lists of all relevant studies were manually reviewed for additional publications.

### Eligibility criteria

We included studies assessing the impact of post-thaw embryo culture on FET outcomes compared to immediate transfer at various developmental stages. The interventions were categorized into four distinct protocols: (A) D2ext: day-2 embryos with extended culture versus immediate transfer; (B) D3ext: day-3 embryos with extended culture versus immediate transfer; (C) D3-to-D5/6: day-3 embryos with extended culture to blastocyst stage versus immediate blastocyst transfer; and (D) D5/6ext: day-5/6 embryos with extended culture versus immediate transfer.

Extended culture was defined as post-thaw incubation periods of 18–48 h before transfer, while immediate transfer was defined as transfer within 2–6 h post-thaw following basic viability assessment. For the day-3 to blastocyst protocol, extended culture specifically referred to culturing thawed day-3 embryos until they reached blastocyst stage (typically day 5 or 6) before transfer.

Studies were eligible if they reported at least one of the following outcomes: implantation rate, positive hCG test, clinical pregnancy rate, live birth rate, or pregnancy loss (biochemical or clinical). We included human studies published in English without initial date restrictions. However, to ensure relevance to current clinical practice, this meta-analysis included only studies using vitrification for embryo cryopreservation. During the screening process, we observed that studies utilizing vitrification were predominantly published from 2010 onwards, as this period marked the widespread adoption of vitrification techniques over slow freezing methods in clinical practice. Consequently, the included studies spanned from 2010 to 2023. Eligible study designs included randomized controlled trials, prospective and retrospective cohort studies, and case–control studies.

We excluded studies involving slow-frozen embryos, studies comparing outcomes of embryos transferred at different developmental stages without post-thaw culture comparison, case reports, case series, reviews, editorials, and letters to the editor. Studies that did not provide sufficient information to assess methods or data for meta-analysis were also excluded. Duplicates were removed prior to screening articles by their title and abstracts.

### Study selection

Titles and abstracts of identified studies were independently screened by two reviewers (ACP and LTM) using the Rayyan platform. When immediate exclusion based on title and abstract was not possible, the full text was assessed. Any disagreement over eligibility was resolved by consultation with a third author (YB). For inclusion, studies had to report both post-thaw culture protocols and relevant FET outcomes.

### Data extraction

Data were extracted by two independent reviewers (ACP and LTM) using a standardized data collection form. The extracted information included the following: author’s name, publication year, country, study design, sample size, embryo development stage at freezing, post-thaw embryo culture protocol and duration, study outcomes, and exclusion criteria. For binary outcomes, we extracted the number of events and total participants in each group from the included studies when available or calculated these values from reported percentages and sample sizes to enable meta-analysis.

Reference lists of included studies were manually reviewed to identify additional potentially relevant publications. In cases where required data were not fully reported in the identified studies, we attempted to contact the original authors via email; however, these attempts did not yield additional data for inclusion in the meta-analysis.

### Outcome measures

The outcome measures were defined based on the included studies’ reporting standards. Clinical pregnancy rate was defined as pregnancies with ultrasound confirmation of gestational sac(s), expressed per embryo transfer. Live birth rate was calculated as deliveries resulting in at least one live-born infant, expressed per embryo transfer. Implantation rate was determined as the number of gestational sacs observed divided by the number of embryos transferred. Clinical miscarriage rate represented spontaneous pregnancy losses after clinical pregnancy confirmation. Biochemical pregnancy loss referred to pregnancies diagnosed by positive hCG that failed to progress to clinical pregnancy. Embryo loss rate was calculated as the proportion of embryos that failed to survive the post-thaw culture period [26].

### Assessment of risk of bias

The methodological quality of each study was assessed independently by two reviewers (ACP and LTM) using the Newcastle–Ottawa Scale (NOS) for cohort and case–control studies [27]. Given the predominance of observational cohort studies in our analysis, we applied the NOS cohort assessment tool across all included observational studies for consistency. For these studies, the NOS assessed three domains: selection of study groups (4 points), comparability of groups (2 points), and assessment of outcome (3 points), with studies scoring ≥ 7 points considered high quality. Any disagreements in quality assessment were resolved through discussion and consensus.

### Data synthesis

We analyzed data according to the four distinct post-thaw culture protocols described in the eligibility criteria. For each protocol, we evaluated multiple outcomes including implantation rate, clinical pregnancy rate, live birth rate, and pregnancy loss rates.

All meta-analyses were conducted using random-effects models to account for potential between-study heterogeneity. Pooled relative risks (RRs) with 95% confidence intervals (CIs) were calculated. Heterogeneity was assessed using the *I*^2^ statistic, with values of 25%, 50%, and 75% representing low, moderate, and high heterogeneity, respectively [28], and tau-squared (*τ*^2^) to quantify between-study variance.

Sensitivity analyses were performed by iteratively removing one study at a time (leave-one-out) and recalculating the summary effect to assess the sources of heterogeneity between studies and to evaluate the robustness of our pooled effect estimates. The stability of results was evaluated by examining changes in point estimates, confidence intervals, and statistical significance. Funnel plots were generated to visually inspect for potential publication bias (Supplementary Figure S3), though interpretation was limited by the small number of studies per comparison (3–4 studies). All analyses were conducted using Comprehensive Meta-Analysis version 4.0 (Biostat Inc., Englewood, NJ, USA). All statistical tests were two-sided, and *p* < 0.05 was considered statistically significant.

## Results

### Study selection and characteristics

**Fig. 1:**
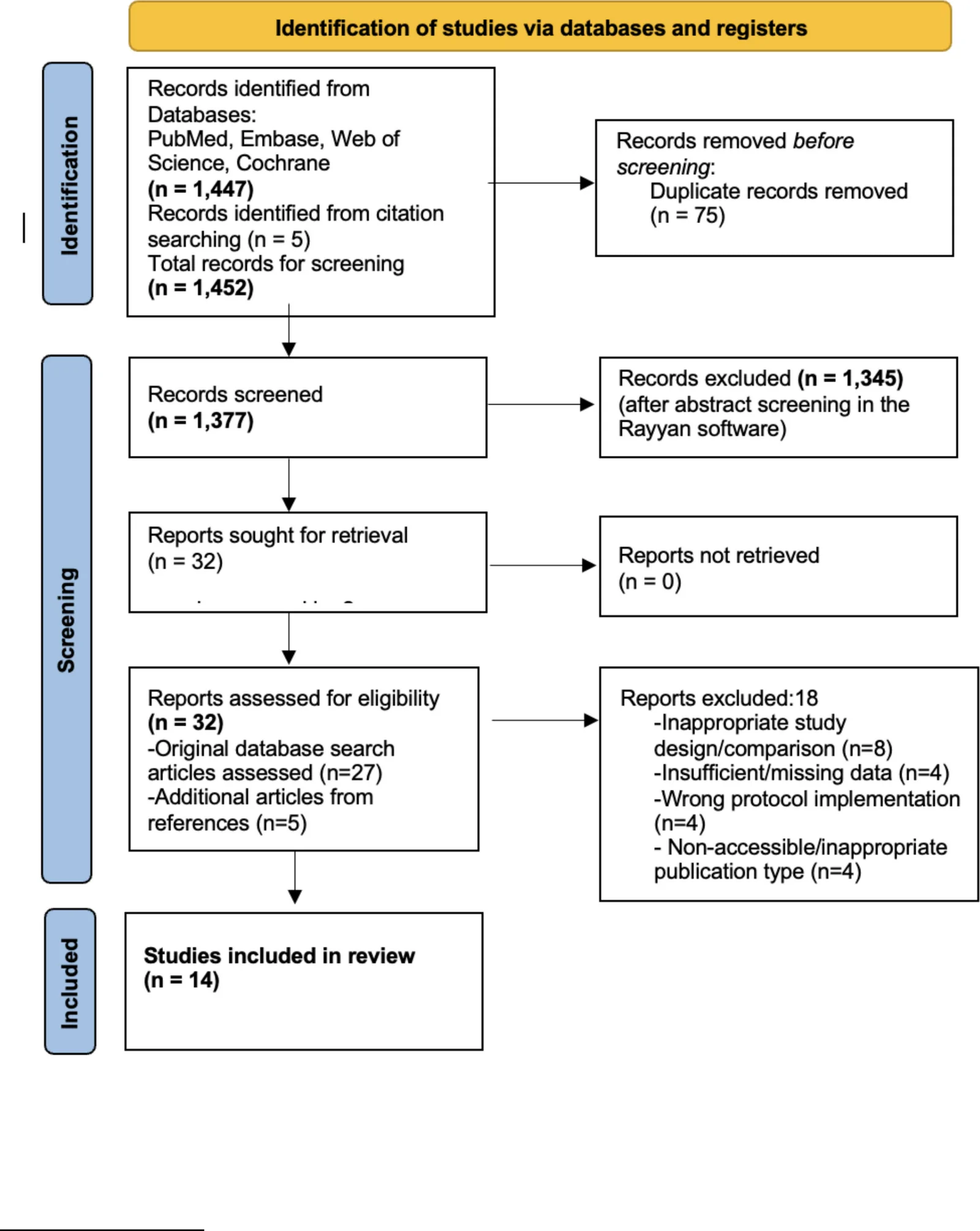
PRISMA flow diagram of study selection process. The flow diagram depicting the systematic search and selection process according to PRISMA 2020 guidelines

After comprehensive screening of 1377 records (following removal of 75 duplicates from 1452 initially identified records), 32 full-text articles were retrieved and assessed for eligibility. Of these, 18 were excluded for inappropriate study design or comparison (*n* = 8), insufficient or missing data (*n* = 4), wrong protocol implementation (*n* = 4), and non-accessible or inappropriate publication type (*n* = 4). Fourteen studies ultimately met the inclusion criteria and were included in the final meta-analysis (Fig. 1). The included studies encompassed 17,415 frozen embryo transfer cycles conducted between 2010 and 2023 across multiple countries including China, Israel, Turkey, Brazil, Vietnam, India, Switzerland, France, and Korea. Study designs included two randomized controlled trials (one of which was prospective interventional), one additional prospective interventional study, and eleven retrospective cohort studies. Extended culture durations ranged from 18 to 48 h across studies (Table 1).

**Table 1:** Characteristics of included studies in systematic review and meta-analysis of post-thaw culture duration effects on frozen embryo transfer outcomes, stratified by embryo developmental stage (*n* = 14 studies)

First author (year of publication)	Study design	Location	Years	Number of subjects (cultured, non-cultured)	Outcomes measured	Exclusion
**Day 2 vs. day 2 extended**						
L. Colodetti (2020) [37]	RCT double-blind	Brazil	2017–2018	388 (209, 179)	Embryo loss, clinical pregnancy, ongoing pregnancy, clinical miscarriage	RPL, implantation failure, severe male factor, uterine pathology, re-vitrified embryos, difficult embryo transfer
M.T. Le (2019) [38]	Prospective cohort study	Vietnam	2016–2018	324 (162, 162)	Transferred embryos, implantation rate, clinical pregnancy, biochemical pregnancy loss	Genetic donor cycles, female with endometriosis, low ovarian reserve, surgical sperm retrieval
G. G. Ranji (2023) [29]	Retrospective comparative	India	2017–2021	167 (75, 92)	Clinical pregnancy	None
**Day 3 to day 3 extended**						
J.Y.Wang (2023) [15]	Retrospective cohort	China	2018–2022	470 (235, 235)	Transferred embryos, implantation rate, clinical pregnancy, ongoing pregnancy, live birth rate, clinical miscarriage	Not first FET cycle, severe uterine anomalies, untreated hydrosalpinx, RPL, diabetes, abnormal thyroid function
Y. Li (2022) [16]	Retrospective cohort	China	2016–2019	9381 (3599, 5782)	Transferred embryos, implantation rate, clinical pregnancy, live birth rate, clinical miscarriage	Women with any uterine anomalies, thawed embryos with damaged blastomeres, transfer of 1 or 3 embryos
L. Guo (2013) [23]	Retrospective cohort	China	2010–2012	1353 (566, 787)	Transferred embryos, implantation rate, clinical pregnancy, live birth rate, clinical miscarriage	None
**Day 3 to 5 vs. day 5–6**						
R. Rahav-Koren (2021) [17]	Retrospective observational cohort	Israel	2014–2020	450 (224, 226)	Positive HCG, clinical pregnancy, ongoing pregnancy, biochemical pregnancy loss, clinical miscarriage	Age > 45 years, fertility preservation, surrogacy, egg donation
G. Onalan (2023) [18]	Retrospective cohort	Turkey	2017–2020	151 (103, 48)	Positive HCG, clinical pregnancy, live birth rate, biochemical pregnancy loss, clinical miscarriage	None
X. Li (2023) [19]	Retrospective cohort	China	2018–2020	214 (48, 166)	Embryo loss, transferred embryos, implantation rate, positive HCG, clinical pregnancy, live birth rate, clinical miscarriage, positive HCG, clinical pregnancy, live birth rate, biochemical pregnancy loss, clinical miscarriage	~ 2 cryopreserved embryos, hydrosalpinx, endometriosis, uterine fibroids
P. C. Aytac (2022) [20]	Retrospective cohort	Turkey	2014–2016	634 (355, 279)		PGT, recurrent implantation failure, recurrent IVF failure, age ~ 40 years
**Day 5–6 vs. day 5–6 extended**						
H. Ji (2023) [14]	Retrospective cohort	China	2019–2021	1639 (711, 928)	Embryo loss, transferred embryos, implantation rate, clinical pregnancy, live birth rate, clinical miscarriage	Age ~ 42, uterine anomalies, cycles of oocyte donation or vitrified oocytes, PGT, complete D3 and D5 blastocyst, had blastocyst previous cycle, incomplete data records
M. Ciaffaglione (2022) [39]	Retrospective observational cohort	Switzerland	2014–2021	1914 (1029, 885)	Embryo loss, transferred embryos, implantation rate, clinical pregnancy, live birth rate, clinical miscarriage	none
Herbemont (2018) [40]	Prospective interventional randomized study	France	2015–2017	162 (81, 81)	Transferred embryo, embryo loss, implantation rate, clinical pregnancy, ongoing pregnancy, clinical miscarriage	Double blastocysts, thin endometrium < 5.8 mm, procedural difficulties
Hwang (2020) [22]	Retrospective cohort	Korea	2017–2018	168 (112, 56)	Transferred embryo, embryo loss, implantation rate, clinical pregnancy, ongoing pregnancy, clinical miscarriage	Double blastocysts, thin endometrium < 8 mm, procedural difficulties

*RCT*, randomized controlled trial; *RPL*, recurrent pregnancy loss; *FET*, frozen embryo transfer; *HCG*, human chorionic gonadotropin; *PGT*, preimplantation genetic testing; *IVF*, in vitro fertilization

The studies were categorized according to four distinct post-thaw culture protocols: (A) D2ext: day 2 extended versus immediate day 2 transfer (3 studies, *N*= 879); since there was only one study in which day-2 embryos were cultured to day 3 [29], a culture duration 6 h longer than the extended day 2 culture (up to 18 h) we decided to include it in this group. (B) D3ext: day 3 extended versus immediate day 3 transfer (3 studies, *N* = 11,204); (C) D3-to-D5/6: day 3 extended to blastocyst-stage versus immediate blastocyst transfer (4 studies, *N* = 1449); (D) D5/6ext: day 5/6 extended versus immediate day 5/6 transfer (4 studies, *N* = 3883). These protocols were analyzed separately to provide clinically relevant guidance for different embryonic developmental stages and optimize frozen embryo transfer outcomes.

### Study quality assessment

**Fig. 2:**
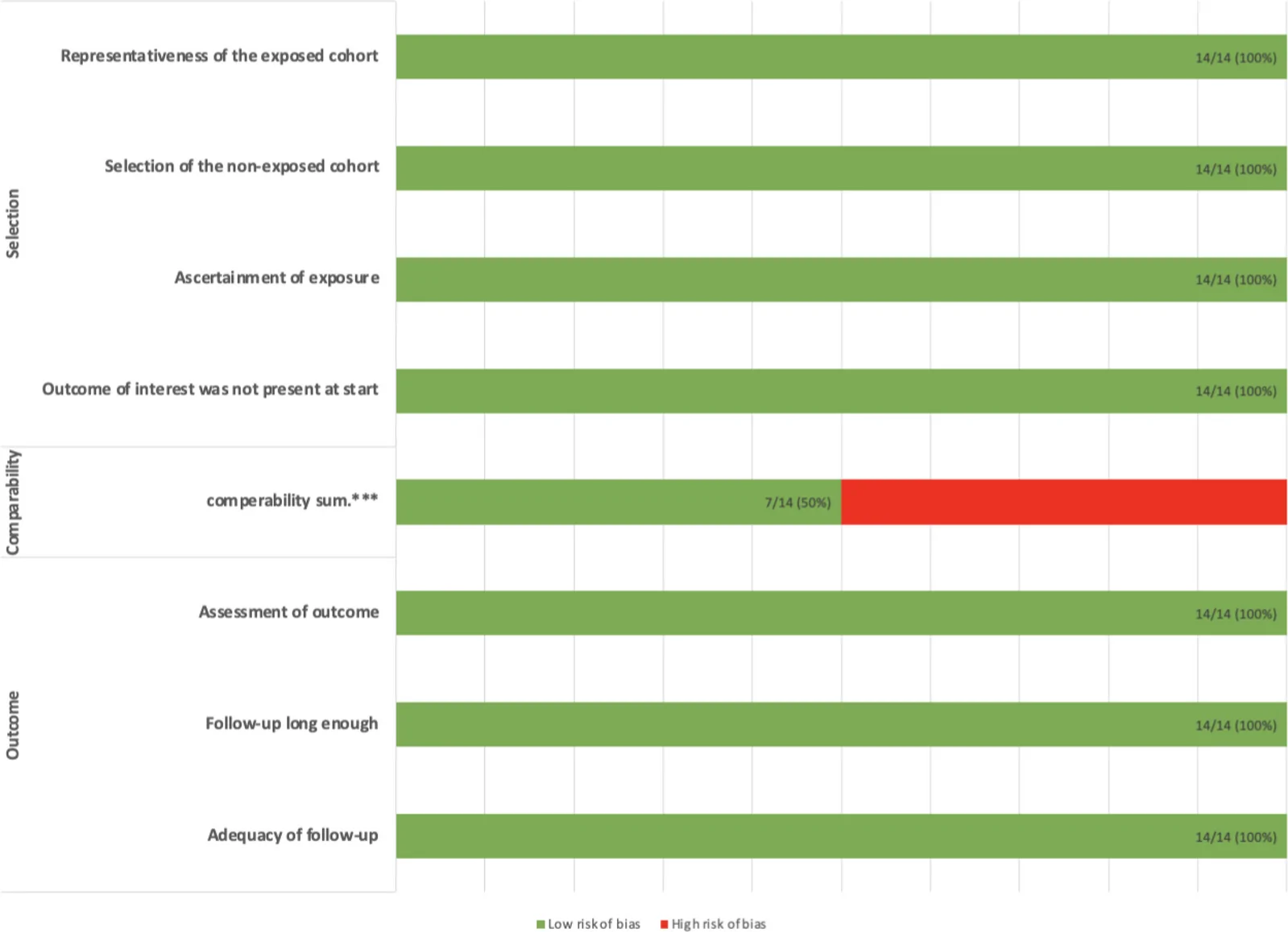
Newcastle–Ottawa Scale risk of bias assessment for all 14 included studies (12 cohort studies and 2 randomized controlled trials). Assessment domains: selection, comparability, and outcome. Risk of bias assessment for all fourteen included studies using modified Newcastle–Ottawa Scale criteria. Green bars indicate low risk of bias, red bars indicate high risk of bias. Assessment categories include: representativeness of exposed cohort, selection of non-exposed cohort, ascertainment of exposure, outcome not present at study start, comparability summary, assessment of outcome, follow-up duration adequacy, and adequacy of follow-up. Numbers and percentages represent the proportion of studies meeting each quality criterion

Risk of bias assessment revealed that the included studies demonstrated generally high methodological quality across key domains (Fig. 2). All fourteen studies (100%) showed adequate representativeness of exposed cohorts, appropriate selection of non-exposed cohorts, and proper ascertainment of exposure. Assessment of outcomes and follow-up adequacy was achieved in all studies (100%). However, comparability assessment revealed notable limitations, with only seven studies (50%) demonstrating adequate control for confounding factors, while the remaining seven studies (50%) had high risk of bias in this domain. Despite this limitation in comparability, the overall quality assessment across other domains supported the reliability of the included studies for the pooled analyses. Between-study heterogeneity was low across most outcomes, with *I*^2^ = 0% (*τ*^2^ = 0.000). Blastocyst-stage extended culture analyses showed moderate to substantial heterogeneity (*I*^2^ = 46–74%).

### Clinical outcomes by embryonic stage

#### D2ext: immediate transfer preferred

For day-2 embryos, extended culture was associated with significantly lower clinical pregnancy rates compared to immediate transfer (Supplementary Figure S1). The analysis of three studies (Le 2019, Colodetti 2020, and Ranji 2023) revealed that clinical pregnancy rates were significantly lower with extended culture (RR = 0.689, 95% CI 0.569–0.835, *p* < 0.001, *τ*^2^ = 0.000, *I*^2^ = 0.0%) based on random-effects meta-analysis. This finding suggests that day-2 embryos may derive greater clinical benefit from immediate transfer rather than prolonged in vitro culture. Sensitivity analysis excluding non-retrospective studies was not feasible for this comparison, as it would leave only one study, which is insufficient for meta-analysis.

### D3ext: benefits from extended culture

**Fig. 3:**
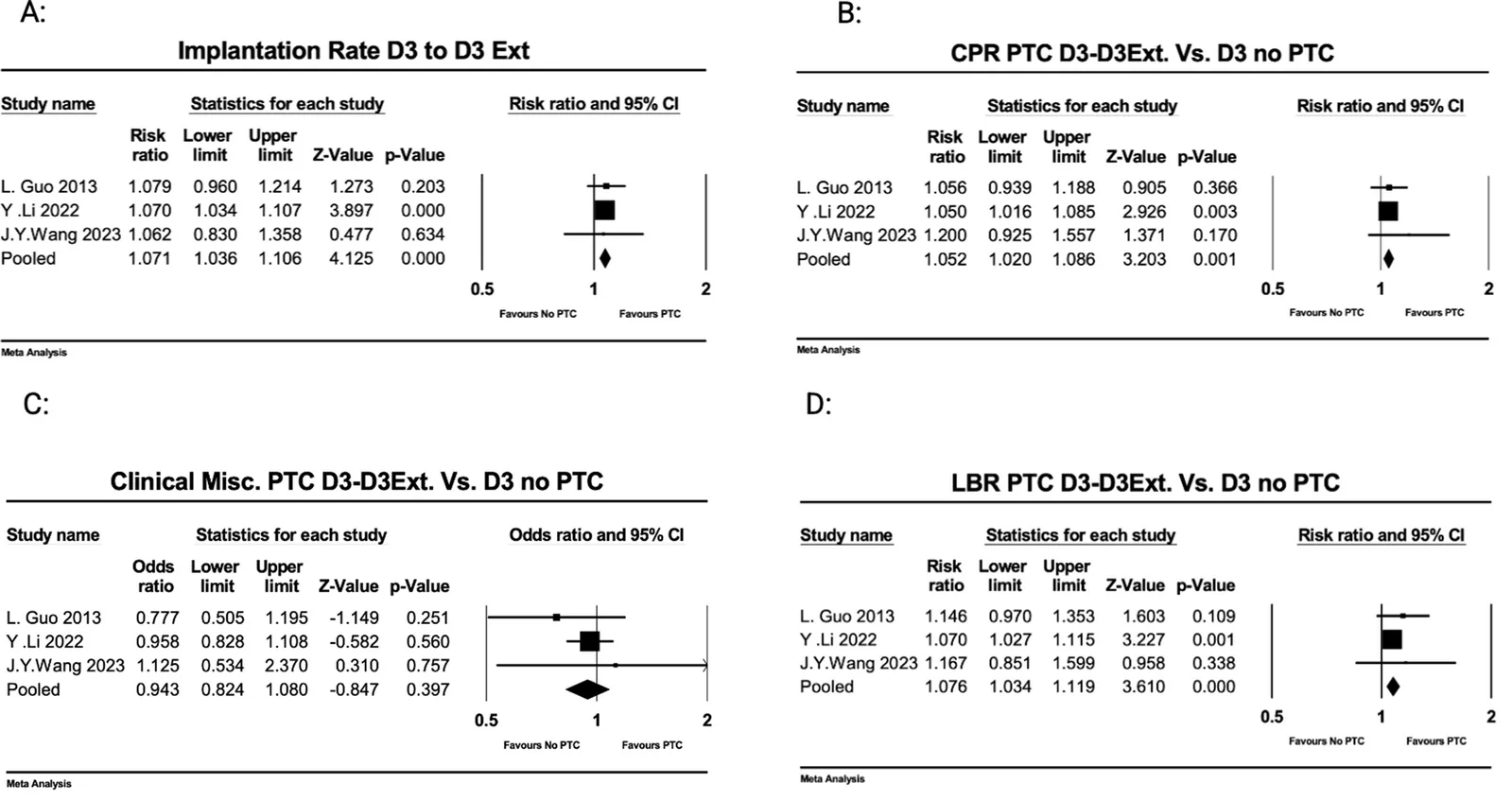
D3ext: Forest plots comparing extended culture versus immediate transfer for day-3 embryos. Forest plots showing relative risks (RR) and odds ratios (OR) with 95% confidence intervals (CI) for day-3 embryo outcomes comparing extended culture to immediate transfer. **A** Implantation rate. **B** Clinical pregnancy rate. **C** Clinical miscarriage rate. **D** Live birth rate. Studies included: Guo 2013, Li 2022, and Wang 2023. Square size reflects study weight in meta-analysis. Diamond represents pooled effect estimate

In contrast to day-2 embryos, day-3 embryos showed significant improvements across multiple reproductive outcomes when subjected to extended culture (Fig. 3). Extended culture significantly enhanced implantation rates (RR = 1.071, 95% CI 1.036–1.106, *p* < 0.001, *τ*^2^ = 0.000, *I*^2^ = 0.0%) based on random-effects meta-analysis of three studies (Guo 2013, Li 2022, and Wang 2023), indicating a 7% relative improvement in implantation success.

Patients undergoing extended culture of day-3 embryos experienced significantly higher clinical pregnancy rates (RR = 1.052, 95% CI 1.020–1.086, *p* = 0.001, *τ*^2^ = 0.000, *I*^2^ = 0.0%) in random-effects meta-analysis, representing a 5% relative improvement in pregnancy achievement. Extended culture significantly improved live birth rates (RR = 1.076, 95% CI 1.034–1.119, *p* < 0.001, *τ*^2^ = 0.000, *I*^2^ = 0.0%) in random-effects meta-analysis, representing approximately a 7% increase in the likelihood of achieving a live birth.

Crucially, extended culture did not increase pregnancy loss rates, with no significant difference observed in clinical miscarriage rates compared to immediate transfer (OR = 0.943, 95% CI 0.824–1.080, *p* = 0.397, *τ*^2^ = 0.000, *I*^2^ = 0.0%) in random-effects meta-analysis. This finding suggests that the improved outcomes were achieved without compromising pregnancy safety.

### D3-to-D5/6: enhanced clinical benefits

**Fig. 4:**
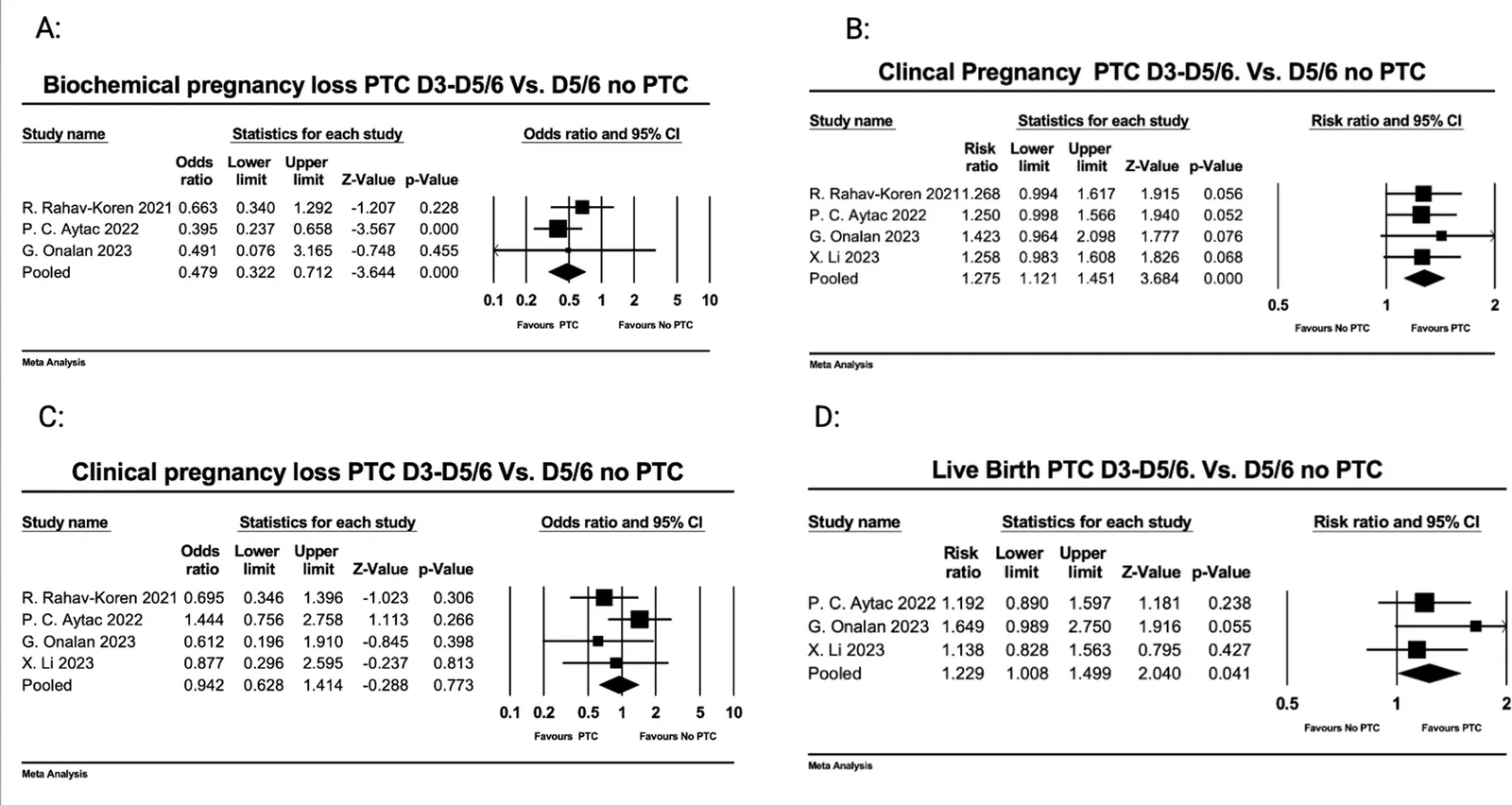
D3-to-D5/6: Forest plots comparing extended culture to blastocyst-stage versus immediate blastocyst transfer for day-3 embryos. Forest plots showing relative risks (RR) and odds ratios (OR) with 95% confidence intervals for day-3 embryos cultured to blastocyst-stage versus immediate day 5/6 blastocyst transfer. **A** Biochemical pregnancy loss. **B** Clinical pregnancy rate. **C** Clinical pregnancy loss. **D** Live birth rate. Studies included the following: Rahav-Koren 2021, Aytac 2022, Onalan 2023, and Li 2023. Square size reflects study weight in meta-analysis. Diamond represents pooled effect estimate

When day-3 embryos were cultured to the blastocyst stage and compared to direct day 5/6 blastocyst transfer, the clinical advantages were pronounced, supported by four comprehensive studies (Rahav-Koren 2021, Aytac 2022, Onalan 2023, and Li 2023) (Fig. 4). The clinical pregnancy rate showed substantial improvement with extended culture (RR = 1.275, 95% CI 1.121–1.451, *p* < 0.001, *τ*^2^ = 0.003, *I*^2^ = 9.0%) based on random-effects meta-analysis, with all four studies demonstrating consistent positive trends. This represents approximately a 27% relative improvement in clinical pregnancy achievement.

Extended culture resulted in significantly higher live birth rates (RR = 1.229, 95% CI 1.008–1.499, *p* = 0.041, *τ*^2^ = 0.000, *I*^2^ = 0.0%) based on random-effects meta-analysis, based on analysis of three studies (Aytac 2022, Onalan 2023, and Li 2023), indicating a 23% relative improvement in the ultimate clinical goal of live birth. Additionally, extended culture was associated with significantly reduced biochemical pregnancy loss (OR = 0.479, 95% CI 0.322–0.712, *p* < 0.001, *τ*^2^ = 0.000, *I*^2^ = 0.0%) based on random-effects meta-analysis, based on three studies (Rahav-Koren 2021, Aytac 2022, and Onalan 2023). However, no significant difference was observed in clinical pregnancy loss rates (OR = 0.942, 95% CI 0.628–1.414, *p* = 0.773, *τ*^2^ = 0.000, *I*^2^ = 0.0%) based on random-effects meta-analysis across four studies, suggesting that the primary benefit lies in improved implantation quality rather than reduced clinical miscarriage after successful implantation.

#### D5/6ext: no additional clinical benefit

For embryos that had already reached the blastocyst stage, extended culture provided no clinical advantage compared to immediate transfer (Supplementary Figure S2). Analysis of three studies (Herbemont 2018, Ciaffaglione 2022, and Ji 2023) revealed no significant differences in embryo loss rates (OR = 1.543, 95% CI 0.541–4.402, *p* = 0.418 *τ*^2^ = 0.578, *I*^2^ = 74.4%) based on random-effects meta-analysis. Live birth rates showed no significant difference (RR = 1.004, 95% CI 0.858–1.175, *p* = 0.958, *τ*^2^ = 0.011, *I*^2^ = 61.8%) based on random-effects meta-analysis. Similarly, analysis of four studies (Herbemont 2018, Hwang 2020, Ciaffaglione 2022, and Ji 2023) showed no significant impact on clinical miscarriage rates (RR = 1.164, 95% CI 0.915–1.481, *p* = 0.216, *τ*^2^ = 0.000, *I*^2^ = 0.0%) based on random-effects meta-analysis. Sensitivity analysis excluding the prospective RCT confirmed these findings.

### Sensitivity analysis and publication bias assessment

Leave-one-out sensitivity analyses were conducted for all primary outcomes to assess the robustness of findings. Sequential removal of individual studies did not materially alter the pooled effect estimates, direction of effects, or statistical significance for any comparison across all embryonic stages. The confidence intervals remained stable, and p-values showed minimal variation when excluding any single study. This confirms the stability and reliability of the meta-analysis results, indicating that no single study was driving the overall conclusions, and the findings were consistent across different study combinations. Funnel plots for all comparisons are provided in Supplementary Figure S3.

## Discussion

### Main findings and biological overview

This meta-analysis demonstrated distinct, stage-specific effects of post-thaw culture duration on frozen embryo transfer outcomes. Most notably, our findings reveal that day-2 embryos achieve significantly better clinical pregnancy rates (RR = 0.689) when subjected to immediate transfer rather than extended in vitro culture. This highlights a critical vulnerability in early-stage cleavage embryos, suggesting that prolonged post-thaw culture is detrimental at this specific developmental stage. In contrast, extended culture provides substantial benefits for day-3 embryos. Extended culture of day-3 embryos significantly improved implantation rates by 7%, clinical pregnancy rates by 5%, and live birth rates by 7%. When day-3 embryos were cultured to the blastocyst stage, the benefits were even more pronounced, with clinical pregnancy rates improving by 27% and live birth rates by 23%. Finally, for embryos already established at the blastocyst stage, additional post-thaw culture provided no clinical advantage (RR = 1.004 for live birth). Collectively, these findings strongly argue against universal “one-size-fits-all” post-thaw protocols, emphasizing the need for stage-tailored strategies.

It is important to clearly distinguish between our two blastocyst-stage comparisons. Protocol C evaluated embryos frozen at the cleavage stage (day 3), thawed, and cultured to the blastocyst stage before transfer, compared to a control group of embryos originally frozen at the blastocyst stage. In contrast, Protocol D evaluated embryos already frozen at the blastocyst stage, assessing whether additional post-thaw culture prior to transfer provided any benefit.

Despite current practice favoring blastocyst-stage embryo cryopreservation and transfer, still, substantial numbers of cleavage-stage embryos are currently preserved worldwide. The absence of consensus guidelines regarding the need for post-thaw culture and its optimal duration represents a significant clinical challenge. Current practice heterogeneity reflects this knowledge gap, with conflicting study findings likely attributable to differences in embryonic developmental stages, study populations, cryopreservation technique and other laboratory conditions, and embryo culture protocols.

The biological rationale underlying our findings may relate to two key factors: embryonic genome activation timing and the limited contribution of post-thaw culture to embryo selection at earlier developmental stages.

Human embryonic development follows a precisely orchestrated timeline, with fertilized embryos progressing through cleavage divisions to reach the 4–8 cell stage by day 3 post-fertilization. This developmental window represents a critical transition point marked by major embryonic genome activation (EGA), where embryonic gene expression assumes control from maternally inherited factors [30]. While recent evidence indicates that EGA initiation occurs as early as the one-cell stage, the major wave of genome activation at day 3 constitutes the most significant developmental checkpoint, where embryos with genetic abnormalities frequently arrest [31, 32]. This biological transition provides the rationale for extended embryo culture, allowing natural selection of developmentally competent embryos while enabling progression to more advanced stages [31, 32]. The incorporation of post-thaw extended culture into the management of cleavage-stage embryos enables more accurate selection of developmentally competent embryos, specifically those that have successfully traversed embryonic genome activation (EGA) and demonstrate appropriate subsequent development. This mechanism may also explain why brief culture of day-2 embryos for only one additional day does not improve pregnancy rates, as this interval does not adequately capture the developmental consequences of EGA or reliably discriminate embryos with true developmental competence. However, this practice entails substantial clinical and laboratory drawbacks that must be acknowledged. Culturing thawed day-3 embryos to the blastocyst stage often requires thawing multiple cleavage-stage embryos simultaneously to ensure at least one viable blastocyst is available for transfer, as described by Li et al. Consequently, surplus embryos that successfully reach the blastocyst stage must undergo re-vitrification. Re-vitrification exposes embryos to additional cryoprotectant toxicity and osmotic stress, potentially decreasing their subsequent viability. Furthermore, this strategy introduces a higher workload for the embryology laboratory, increases resource utilization and costs, and inherently carries a risk of cycle cancellation if no embryos survive to the blastocyst stage. Extended culture following thawing appears to approximate conditions closer to those of fresh embryos through a “rejuvenation” effect. The cryopreservation process, despite high survival rates with vitrification, inevitably introduces cellular stress that may temporarily compromise embryonic function. Extended culture provides a recovery period during which embryos can reestablish normal physiological processes, restoring cellular functions, membrane integrity, and metabolic competence that determine implantation potential [33, 34].

Our findings are consistent with these integrated biological mechanisms: only embryos capable of successfully navigating genome activation and recovering from cryopreservation stress reach the blastocyst stage for transfer [31, 32].

Extended embryo culture is associated with substantial developmental attrition, the majority of which occurs during subsequent in vitro development rather than as an immediate consequence of the warming procedure itself. Contemporary vitrification protocols achieve post-warming survival rates of approximately 90–96%, indicating that only about 4–10% of embryos are lost at the time of thaw [5, 7]. However, when cleavage-stage embryos undergo extended culture, a considerably larger proportion fail to advance to later developmental stages.

The studies included in this meta-analysis did not report cumulative pregnancy rates per cycle, precluding a precise comparison of overall attrition between the culture–freeze–transfer strategy and the shorter culture–freeze–post-thaw culture–transfer approach. Furthermore, while significant attrition is biologically expected when culturing cleavage-stage embryos to the blastocyst stage, there is a notable lack of reporting regarding embryo attrition in studies evaluating shorter extended culture periods (e.g., day 2 extended or day 3 extended). In these specific cohorts, it is difficult to ascertain whether the absence of documented embryo loss reflects a true 100% developmental progression or simply under-reporting by the original authors.

Nevertheless, the data summarized in Supplementary Table S1 provide information on embryo loss in cohorts intended either for direct thaw-and-transfer or for post-thaw extended culture prior to transfer. The apparent attrition rates in these groups are broadly comparable. Consistent with this observation, multiple studies assessing the developmental competence of day-3 embryos report that only approximately 30–50% progress to the blastocyst stage, implying that 50–70% arrest during extended culture [35, 36]. This attrition reflects intrinsic embryonic competence and the natural selection processes associated with embryonic genome activation and blastulation. Accordingly, most embryo loss observed in extended culture protocols is attributable to developmental arrest rather than to cryopreservation injury or suboptimal culture conditions [31, 35]. Furthermore, the superior clinical outcomes reported after post-thaw extended culture may partly result from the preferential exclusion of embryos with lower developmental potential, thereby enriching for embryos with greater capacity for continued development.

The results of our study show that blastocyst-stage embryos demonstrate no benefit from extended culture, likely because they have already passed the critical genome activation checkpoint and achieved developmental competence. These embryos possess established metabolic machinery and cellular organization that render additional culture time unnecessary and potentially detrimental through exposure to suboptimal in vitro conditions.

### Strengths and limitations

This meta-analysis provides the most comprehensive systematic assessment to date of post-thaw culture strategies, synthesizing evidence from 14 studies encompassing more than 17,000 transfer cycles. Strengths include comprehensive searches and protocol-specific stratification, as well as conservative random-effect model approach. Stratifying results according to embryonic stage and focusing exclusively on vitrified embryos provides clinically relevant and contemporary insights.

However, several limitations warrant consideration, primarily stemming from the design and reporting practices of the primary studies. First, our analysis strictly evaluates outcomes per embryo transfer and does not account for cycle cancellation rates. This is a crucial limitation, as patients undergoing extended culture face a real clinical risk of having no embryos available for transfer. Second, there is incomplete and inconsistent reporting of embryo attrition across the included studies. While attrition from the cleavage to the blastocyst stage is physiologically expected, there is a notable lack of attrition data in studies evaluating shorter extended culture periods (e.g., day 2 or day 3 extended). This potential under-reporting makes it difficult to ascertain true developmental progression versus reporting bias, thereby limiting our ability to accurately estimate cumulative pregnancy rates per patient. Furthermore, for day-2 embryos specifically, the available literature only permitted the meta-analysis of clinical pregnancy rates, limiting our ability to draw definitive conclusions regarding live birth or miscarriage rates for this cohort.

Third, several unmeasured confounders and potential biases within the primary literature must be acknowledged. Many included studies involved the transfer of multiple embryos, and the average number of embryos transferred was not consistently adjusted for across comparisons. Additionally, when comparing day-3 embryos cultured to blastocyst versus direct blastocyst thaw, it is difficult to ascertain whether the highest-quality blastocysts were preferentially thawed and transferred first in the control groups, which could introduce significant selection bias. Variations in endometrial preparation protocols (e.g., natural versus artificial cycles) were also not uniformly controlled for across studies, which could impact optimal embryo-endometrial synchronicity and serve as an unmeasured confounder.

Finally, limitations inherent to the meta-analysis itself must be noted. The limited number of studies per comparison group (3–4 studies) restricts statistical power, particularly for detecting modest effects or confirming null associations, and precludes the formal statistical assessment of publication bias. Additionally, while statistical heterogeneity was generally low across the analysis, moderate heterogeneity was observed in the blastocyst-stage comparisons (*I*^2^ = 46–74%). This, combined with the inherent clinical heterogeneity in culture media, laboratory practices, and baseline patient characteristics across different fertility centers, may affect the overall generalizability of these findings.

Future research should consistently document embryo loss during culture and adopt standardized protocols to improve comparability and refine clinical recommendations. An important methodological limitation is that several studies did not adjust for key confounding variables, such as maternal age, embryo quality metrics, or endometrial preparation protocols. This lack of adjustment reduces confidence in the extent to which the observed associations can be attributed solely to culture duration. Additionally, the absence of explicit exclusion criteria in some studies may have produced heterogeneous study populations, making it more difficult to identify subgroups who could benefit most from extended culture.

Another consideration relates to quality assessment. Although the review included two randomized controlled trials alongside multiple observational cohorts, we applied the Newcastle–Ottawa Scale uniformly to all observational studies for consistency. While this approach supports comparative evaluation across studies, it may not fully capture methodological nuances unique to different study designs.

## Conclusions

This meta-analysis provides evidence that may inform guidance for post-thaw culture strategies. Our findings suggest that optimal protocols should be tailored to embryonic developmental stage. Extended culture of day-3 embryos to blastocyst stage shows meaningful clinical benefits, potentially through genome activation-mediated selection and post-thaw recovery mechanisms. However, these findings assume standardized vitrification, warming, and culture protocols across centers. Implementation must consider significant inter-laboratory heterogeneity in culture systems, incubator technology, temperature control precision, media formulations, and embryo handling protocols, all of which may influence post-thaw embryo development and survival. Clinical implementation could consider incorporating mathematical models that account for baseline developmental competence, expected progression rates, and stage-specific survival probabilities within individual laboratory settings. Prolonged culture of same-stage embryos appears to provide limited advantage, likely because it does not advance embryos through important developmental checkpoints.

While implementation of stage-specific protocols could improve per-transfer outcomes for patients with cryopreserved cleavage-stage embryos, these clinical benefits must be carefully weighed against significant potential disadvantages. These include the risk of cycle cancellation, the potential need for embryo re-vitrification, and increased laboratory resource utilization. These findings support the development of stage-tailored approaches that balance the potential clinical benefits of extended culture against laboratory capacity, cost considerations, and patient-specific factors.

## Supplementary Information

Below is the link to the electronic supplementary material.

ESM1 (DOCX 1.08 MB)

## Data Availability

All data generated or analyzed during this study are included in this published article and its supplementary information files.
