# Supplementary material for: The impact of post thaw embryo culture (extended culture) on frozen embryo transfer outcomes: a systematic review and meta-analysis
Source: J Assist Reprod Genet. 2026 Jun 12;43(7):2185–98. doi: 10.1007/s10815-026-03919-w (PMC13518672; doi:10.1007/s10815-026-03919-w)
Supplement: Supplementary file 1 [file 10815_2026_3919_MOESM1_ESM.docx]

# Supplementary Figures

**Supplementary Figure S1. D2ext: Forest plot comparing extended culture versus immediate transfer for day 2 embryos**

Forest plot showing relative risk (RR) and 95% confidence interval for clinical pregnancy rate in day 2 embryos comparing extended culture to immediate transfer. Three studies included: Le 2019, Colodetti 2020, and Ranji 2023. Square size reflects study weight in meta-analysis. Diamond represents pooled effect estimate.

### **Supplementary Figure S2. D5/6ext: Forest plots comparing extended culture versus immediate transfer for day 5/6 blastocyst embryos**

 Forest plots showing relative risks (RR) and odds ratios (OR) with 95% confidence intervals for blastocyst-stage embryos comparing extended culture to immediate transfer. Panel A: Embryo loss. Panel B: Clinical miscarriage rate. Panel C: Live birth rate. Studies included: Herbemont 2018, Ciaffaglione 2022, and Ji 2023.

### **Supplementary Figure S3. Funnel plots for assessment of publication bias.**

Funnel plots examining publication bias for primary outcomes with sufficient studies. Panel A: Day 3 embryo clinical pregnancy rate. Panel B: Day 3 embryo live birth rate. Panel C: Day 3 to blastocyst clinical pregnancy rate. Panel D: Day 3 to blastocyst live birth rate. X-axis represents effect size (relative risk or odds ratio), Y-axis represents standard error (inverted). Each dot represents one study. Vertical line indicates no effect. Note: Interpretation of funnel plot asymmetry is limited by the small number of studies per comparison (3-4 studies).

**Supplementary Table S1. Embryo survival and developmental attrition rates during post-thaw extended culture.**

| **Author** | **comparison groups** | **Cum PR PTC** | **Cum PR no PTC** | **Post thaw attrition PTC** | **Ext culture attrition PTC** | **Post thaw attrition No PTC** |
| --- | --- | --- | --- | --- | --- | --- |
| **L. Colodetti (2020(** | D2 TO D3 | Unreported | Unreported | 4.90% | 0% | 9.70% |
| **M.T. Le (2019)** | D2 TO D3 | Unreported | Unreported | 3.10% | 0% | 5.50% |
| **G. G. Ranji (2023(** | D2 TO D3 | Unreported | Unreported | Unreported | Unreported | Unreported |
| **J.Y.Wang (2023)** | D3 vs D3Ext. | Unreported | Unreported | Unreported | Unreported | Unreported |
| **Y. Li (2022)** | D3 vs D3Ext. | Unreported | Unreported | Unreported | Unreported | Unreported |
| **L. Guo (2013)** | D3 vs D3Ext. | Unreported | Unreported | Unreported | Unreported | Unreported |
| **R. Rahav-Koren (2021)** | 3to5 vs 5 | Unreported | Unreported | Unreported | Unreported | Unreported |
| **G. Onalan (2023)** | D3T3 vs D3T5 vs D5T5 | Unreported | Unreported | Attrition: D3T3 29.26%, D3T5 12.72%, D5T5 15.81% | Unreported | Unreported |
| **X. Li (2023)** | D3T3 vs D3T5 vs D5T5 | Unreported | Unreported | Attrition: D3T3 4.71%, D3T5 5.04%, D5T5 0% | Unreported | Unreported |
| **P. C. Aytac (2022)** | 3to5 vs 5 | Unreported | Unreported | 3.20% | 7.10% | 5% |
| **H. Ji (2023)** | D5toD5ext | Unreported | Unreported | 13/496 2.6% | Unreported | 12/498 2.4% |
|  | D6toD6ext | Unreported | Unreported | 14/501 2.8% | Unreported | 18/799 2.2% |
| **M. Ciaffaglione (2022(** | D5toD5ext | Unreported | Unreported | 8/1029 0.7% | Unreported | 5/885 0.5% |
| **Herbemont (2018)** | D5toD5ext | Unreported | Unreported | 0/115 | Unreported | 1/103 |
| **Hwang (2020**( | D5toD5ext | Unreported | Unreported | Unreported | Unreported | Unreported |
